# Decellularized esophageal tubular scaffold microperforated by quantum molecular resonance technology and seeded with mesenchymal stromal cells for tissue engineering esophageal regeneration

**DOI:** 10.3389/fbioe.2022.912617

**Published:** 2022-10-04

**Authors:** Maurizio Marzaro, Gianantonio Pozzato, Stefano Tedesco, Mattia Algeri, Alessandro Pozzato, Luigi Tomao, Ilaria Montano, Filippo Torroni, Valerio Balassone, Anna Chiara Iolanda Contini, Luciano Guerra, Tommaso D’Angelo, Giovanni Federici di Abriola, Lorenzo Lupoi, Maria Emiliana Caristo, Ivo Boškoski, Guido Costamagna, Paola Francalanci, Giuseppe Astori, Angela Bozza, Andrea Bagno, Martina Todesco, Emanuele Trovalusci, Luigi Dall’ Oglio, Franco Locatelli, Tamara Caldaro

**Affiliations:** ^1^ Pediatric Surgery Department AULSS2 Treviso, Treviso, Italy; ^2^ Telea Electronic Engineering Vicenza, Vicenz, Italy; ^3^ Telea Biotech Vicenza, Vicenz, Italy; ^4^ Department of Pediatric Onco-Hematology and Cell and Gene Therapy, Bambino Gesù Children’s Hospital, IRCCS, Rome, Italy; ^5^ Digestive Endoscopy and Surgical Unit, Bambino Gesù Children’s Hospital, Rome, Italy; ^6^ Cen.Ri.S. Policlinico Gemelli UNICATT Rome, Rome, Italy; ^7^ Fondazione Policlinico Universitario Agostino Gemelli IRCCS, Digestive Endoscopy Unit, Rome, Italy; ^8^ Università Cattolica del Sacro Cuore, Centre For Endoscopic Research Therapeutics and Training (CERTT), Rome, Italy—CERTT Gemelli, Rome, Italy; ^9^ Pathology Department Bambino Gesù Children’s Hospital, Rome, Italy; ^10^ Advanced Cellular Therapy Laboratory, Haematology Unit, San Bortolo Hospital, Vicenza, Italy; ^11^ Consorzio Per la Ricerca Sanitaria (CORIS) of the Veneto Region, Padova, Italy; ^12^ Department of Industrial Engineering, University of Padova, Padova, Italy; ^13^ Pediatric Surgery Department AULSS2 Treviso, University of Padova, Padova, Italy; ^14^ Department of Pediatrics, Sapienza University of Rome, Roma, Italy

**Keywords:** tissue engineering, esophagus, quantum molecular resonance, mesenchymal stromal cells, scaffold

## Abstract

Current surgical options for patients requiring esophageal replacement suffer from several limitations and do not assure a satisfactory quality of life. Tissue engineering techniques for the creation of customized “self-developing” esophageal substitutes, which are obtained by seeding autologous cells on artificial or natural scaffolds, allow simplifying surgical procedures and achieving good clinical outcomes. In this context, an appealing approach is based on the exploitation of decellularized tissues as biological matrices to be colonized by the appropriate cell types to regenerate the desired organs. With specific regard to the esophagus, the presence of a thick connective texture in the decellularized scaffold hampers an adequate penetration and spatial distribution of cells. In the present work, the Quantum Molecular Resonance^®^ (QMR) technology was used to create a regular microchannel structure inside the connective tissue of full-thickness decellularized tubular porcine esophagi to facilitate a diffuse and uniform spreading of seeded mesenchymal stromal cells within the scaffold. Esophageal samples were thoroughly characterized before and after decellularization and microperforation in terms of residual DNA content, matrix composition, structure and biomechanical features. The scaffold was seeded with mesenchymal stromal cells under dynamic conditions, to assess the ability to be repopulated before its implantation in a large animal model. At the end of the procedure, they resemble the original esophagus, preserving the characteristic multilayer composition and maintaining biomechanical properties adequate for surgery. After the sacrifice we had histological and immunohistochemical evidence of the full-thickness regeneration of the esophageal wall, resembling the native organ. These results suggest the QMR microperforated decellularized esophageal scaffold as a promising device for esophagus regeneration in patients needing esophageal substitution.

## Introduction

The vast majority of patients needing an esophageal replacement undergo gastric or intestinal transpositions with a frequently poor residual quality of life, both in adult (Ellis, 1999; Gawad et al., 1999; Kato et al., 2007; Mariette et al., 2007; Khan et al., 2008; Almhanna, 2012; Wang et al., 2020; Xu et al., 2021) and in pediatric age (Ring et al., 1982; Spitz, 1984; Stone et al., 1986; Othersen et al., 1988; Cywes et al., 1993; Ure et al., 1995; Ludman and Spitz, 2003; Bagolan et al., 2004; Uygun, 2015; Arnold and Numanoglu, 2017; Awad and Jaffray, 2017; Chirica et al., 2017).

In recent years, the use of biomaterials helped to hypothesize tailored esophageal substitutes as “self-developing” organs that simplify surgical procedures, improve patients’ quality of life and follow the host’s growth, which is crucial in the pediatric age.

Natural decellularized scaffold’s residual extracellular matrix (ECM) is produced by the resident cells of an organ for structural and functional purposes, and its biochemical and mechanical features vary according to the specific organ and site (Jiang et al., 2021).

Mesenchymal stromal cells (MSCs) seeded scaffolds showed reduced inflammation and increased reconstructive process (Gronthos and Simmons, 1996; Marzaro et al., 2002; Conconi et al., 2005) since only few MSCs engrafted into the scaffold differentiate toward the same histology of proper cells (Rendra et al., 2020), while MSCs main activity lies in the release of bioactive molecules and vesicles mediating anti-inflammatory action, promoting re-epithelialization, angiogenesis and muscle tissue formation that lead to tissue regeneration. All these peculiarities are better expressed by a 3D cell culture (Li et al., 2018).

As evidenced in our previous experimental trials (Marzaro et al., 2006), the 3D natural matrix tight texture ensures tissue resistance and surgical handling but enables only a two-dimensional (2D) cell seeding on the upper surface. This is a limit for MSCs penetration into the deepest layers of the matrix, and represents an issue to be solved to guarantee an effective microenvironment with high numbers, good survival and effective action of the seeded cells.

To address this problem, we applied an original microperforation technique on the decellularized esophageal scaffolds by a robot-guided needle connected to a Quantum Molecular Resonance^®^ (QMR) generator. This equipment produces a particular electric current with quanta of energy, which does not increase the tissue temperature to more than 50°C. Therefore, the energy at the tip of the needle breaks the ECM molecular bonds without burns or damages (Pozzato and Vignato, 2003; JUSTIA, 2022; Patents Telea Biotech, 2022). The robot realizes a regular thick microchannel system inside the scaffold, avoiding changes in its macroscopic structure and allowing a uniform spatial distribution of the cells after seeding.

Aim of the work is the proof of concept of a tissue engineering esophageal substitution. We prepared a full-thickness pig-derived tubular esophageal scaffold to substitute a corresponding tract of the thoracic esophagus in a large animal based experimental model. After the implantation the surviving animals had a 6 month long follow-up period, they had a gastrostomy for the first postoperative days together with an endoluminal stent, were free to go on autonomous oral feeding as soon as possible and underwent regular endoscopic and radiologic controls. After the sacrifice we had demonstration of the full-thickness regeneration of the esophageal wall in the tract where it was substituted by the scaffold. The length of the regenerated tract corresponded to the implanted scaffold, the esophageal lumen was patent and the animals were able to go on autonomous oral alimentation.

The proposed approach is a further step on the way to overcome by tissue engineering techniques the actual problems related to the thoracic transposition of an intestinal segment or the stomach for esophageal substitution.

## Materials and methods

### Site and regulation

The scaffold preparation was carried out at Telea Biotech facilities (Vicenza, Italy), the cell preparation at the Hemato-Oncology laboratory of the Bambino Gesù Children’s Hospital (OPBG, Rome, Italy), and the animal trial at Cen.Ri.S. (Centro Ricerche Sperimentali Università Cattolica, Rome, Italy).

Experiments were performed in compliance with the directive 2010/63/EU on the protection of animals used for scientific purposes and in compliance with the Italian animal welfare and veterinary health rules and regulations. This project was approved by the ethical committee of the Catholic University of Rome Italy and the Italian Ministry of Health, aut. n° 786/2016-PR (answer to prot. 1F295.17, 03-05-2016) and n° 853/2019-PR (answer to prot. 1F295.82).

### Sample procurement

Esophageal samples were taken in sterile conditions from 10 adult heart-beating 40 kg donor pigs (sus scrofa domesticus). These animals were undergoing experimental surgical trials in general anesthesia other than ours and were already destined to be sacrificed. Harvesting the esophagus still in heart-beating conditions gave us the opportunity to avoid post-mortem necrotic and infectious phenomena of the tissues and guarantee all the ECM properties for the subsequent procedures.

Animals assumed water and glucose solution until 8 h before the operation, and then were fasting. Ceftiofur (Excenel) 3 mg/kg/day was administered intramuscularly (1 ml/16 kg for each injection) starting 24 h before the operation. Anesthesia was induced through a venous catheter in the auricular vein with diazepam 0.5 mg/kg and ketamine 1–5 mg/kg or alternatively propofol 1–8 mg/kg, and continued with 2%–3% isoflurane and continuous propofol infusion (2 mg/kg). Muscle relaxation was obtained by Tracrium (Atracurium) infusion 1 mg/kg. Animals were intubated with 4/5.5 mm cuffed tracheotubes and were kept in mechanical ventilation, monitoring blood pressure and blood parameters, using 5% glucose, crystalloids and colloids if necessary.

At the end of the principal surgical procedure the esophagus was reached and removed through a right thoracotomy in left lateral decubitus, the adventitia was removed, the cervical and cardial portions removed avoiding their different muscle structure, and the proximal end identified with a prolene 3/0 suture.

The animals were sacrificed at the end of the procedures by intravenous injection of tanax 3 ml/10 kg.

The whole thoracic esophagus was used as a tube preserved in all its layers other than the adventitia. Samples were rinsed twice in sodium chloride sterile solution (Sigma), to wash both the surface and the inner lumen. Then they were dried with sterile gauze, inserted in plastic vials and stored at −80°C.

### Esophagus decellularization

After transportation in dry ice, samples were thawed at room temperature for 3 h. The tubular scaffolds were then rinsed in ultrapure water and stored in a solution composed of ultrapure water and 2% penicillin-streptomycin (10,000 units penicillin and 10 mg streptomycin per mL in 0.9% NaCl—Sigma-Aldrich) (AF) for 48 h at 4°C in static conditions. Therefore samples were placed five times for 4 h at room temperature in a 4% sodium deoxycholate solution (BioXtra ≥98.0%, Sigma-Aldrich). Then they underwent a five times treatment with 2,000 Kunitz Unit (KU) of DNase-I (Warthington) in 1 M NaCl solution, each time incubated for 3 h at 37°C. Thereafter, they were rinsed again in ultrapure water and, in order to remove the decellularization reagents, were washed with increasing percentages of denatured ethanol (ACS Reagent, ≥99.8%, without additive, Honeywell) and rehydrated in ultrapure water. Scaffolds were finally stored in ultrapure water and 2% AF at 4°C.

Tubular scaffolds underwent a dynamic decellularization method by means of a dedicated bioreactor that allowed the decellularization fluids to circulate in sterile conditions by perfusion both on the luminal mucosal aspect and on the external muscular side of the scaffold.

### QMR perforative treatment

The decellularized scaffold underwent a microscopic perforative treatment (patented by Telea Biotech, subsidiary of Telea Electronic Engineering) through a 150 μm-diameter needle mounted on a 3-axis Cartesian robot handpiece (Yamaha model RCX240) and connected to the QMR based device developed for this application (Pozzato and Vignato, 2003; JUSTIA, 2022; Patents Telea Biotech, 2022).

Samples of tubular full-thickness esophageal decellularized scaffolds derived from the entire cylindrical esophagus were mounted on a cylindrical agarose (Alfa Aesar) based support that preserved the native shape, conductive for electrical current and rotating on its longitudinal axis. This system was equipped with a tray for the liquids required for the irrigation during the procedure. We used three different supports of increasing dimensions to hold esophagi with different diameters. The perforation was performed by the robot following a specific algorithm that guided the needle in the longitudinal direction, from the upper pole to the lower one, and from the outer muscular surface to the inner mucosal layer, until the entire circumference was completed. The needle action was enough to drill the connective tissue for all its thickness and the free spaces between the channels were deemed similar to their diameter.

MilliQ solution (Merck Millipore) was used to keep the connective tissue humid, in class-II biological safety cabinet under aseptic conditions. No final sterilization method was applied since all the steps of the procedure took place in complete sterility.

### DNA extraction and quantification

Double stranded DNA was quantified both in decellularized and control tissue samples using DNeasy Blood and Tissue kit (Qiagen) following manufacturer’s instructions. A maximum of 25 mg of tissue were cut into small pieces and digested overnight at 56°C with Proteinase K in buffer ATL. Samples were transferred into spin columns after addition of ethanol and buffer AL, and centrifuged for 1 min at 8,000 rpm. After two washing steps with Buffer AW1 (8,000 rpm for 1 min) and AW2 (14,000 rpm for 3 min), DNA was eluted by incubation for 1 min with Buffer AE and centrifugation at 8,000 rpm for 1 min. We quantified the DNA by using a BioPhotometer Plus (Eppendorf) at 260 and 280 nm to estimate its purity and yield.

Statistical analysis was performed by one sample *t*-test with GraphPad Prism Software. Statistical significance is reported as *** when *p* ≤ 0.001.

### Histological and immunohistochemical analyses on native and decellularized esophageal scaffolds

Samples of native esophagi and decellularized scaffolds were fixed in formalin and embedded in paraffin. Transversal esophageal 2.5 µm sections were analyzed with hematoxylin and eosin (H&E) and Masson trichrome dyes using an optical microscope BX53 (Olympus Tokyo, Japan). Sections were deparaffinized, subjected to antigen retrieval and blocked in 5% bovine serum albumin (BSA) for 1 h at room temperature. Tissue sections were then incubated with smooth muscle actin, cytokeratin, collagen-I (Sigma-Aldrich), collagen-IV (Biorbyt), laminin (Sigma-Aldrich), fibronectin (Abcam) and elastin (Sigma-Aldrich) primary antibodies in 1% BSA overnight at 4°C. Sections were washed and incubated with fluorophore-conjugated secondary antibodies (Thermo Fisher) for 1 h at room temperature. Following nuclei staining with Hoechst, tissue sections were mounted with Mowiol^®^4-88. Images were acquired with Axiovert 40 CFL (Zeiss) microscope and LasX software. Other samples were fixed with PFA 4% for 30 min, dehydrated in sucrose 30% overnight and cut with cryostat microtome, then 20 μm sections were obtained (Leica 1860 cryostat) and stained with Hoechst in blue for nuclei and green phalloidin (f-actin) for cytoskeletal mark.

### Biomechanical tests

For the biomechanical tests, the following samples were considered: native porcine esophagus (NPE), decellularized porcine esophagus (DPE), and perforated decellularized esophagus (QMR).

All tissues were excised along the longitudinal direction, parallel to the principal axis of the conduit, and adjacent pair of strips were taken in longitudinal and circumferential directions. The dimensions of strips were 25 mm length and 5 mm width.

Samples thickness was measured using a Mitutoyo digital caliper (model ID-C112XB, Aurora, Illinois, United States ): each specimen was sandwiched between two glass slides, subtracting their thickness.

A custom-made apparatus (IRS, Padova, Italy) was used for the uniaxial tensile loading tests. The system is equipped with four linear actuators and four loading cells (50 N). Uniaxial tests were performed using two actuators and 2 cells at room temperature; samples were continuously wetted with 0.9% NaCl solution to prevent dehydration. Samples were preloaded up to 0.1 N, then elongated up to 300% (elongation rate = 0.2 mm/s) to measure the Ultimate Tensile Strength (UTS) and the Failure Strain (FS). Two elastic modules were calculated as the slope of the stress-strain curve in the linear regions: E_1_ within 0%–10% deformation, and E_2_ in the range between 60 and 100%. Engineering stress σ (MPa) was defined as the tensile force (Newton) divided by the original cross-sectional area of the sample; strain ε (%) was defined as the ratio between the grip displacement and the initial length, which has been set at 5 mm.

UTS, FS, E_1,_ and E_2_ parameters were obtained using an in-house developed Matlab^®^ script (Mathworks, Natick, MA, United States), results were expressed as mean ± standard deviation (SD). N = 16 specimens for each kind of tissue have been tested, 8 in the longitudinal and 8 in the circumferential direction.

Group comparison were conducted with one-way ANOVA and significance was set at *p* < 0.05. Graphpad Prism version for MacOS (GraphPad Software, San Diego, California, United States) was used for the statistical analysis.

### Cell cultures, BM-MSCS phenotyping and Adipogenic/Osteogenic differentiation

Bone marrow aspirates were taken from pigs’ tibia and the samples were diluted 1:1 in PBS to be further stratified on Ficoll reagent for the isolation of the mononuclear cells. After centrifugation, cells were washed twice in PBS, counted and plated at a density of about 2 × 10^5^/cm^2^ in ventilated flasks with αMEM medium supplemented with 16% of FBS and 1% antibiotic/antimycotic (penicillin/streptomycin) and L-glutamine. After 48 h, the medium was changed: thereafter, it was replaced 2 times a week until MSCs colony growing was observed. When a confluence of 70%–80% was reached, the MSCs were harvested and plated at a concentration of about 4 × 10^4^/cm^2^. Part of the harvested cells was used for biological and functional characterization to confirm their nature, as described later on.

**FIGURE 1:**
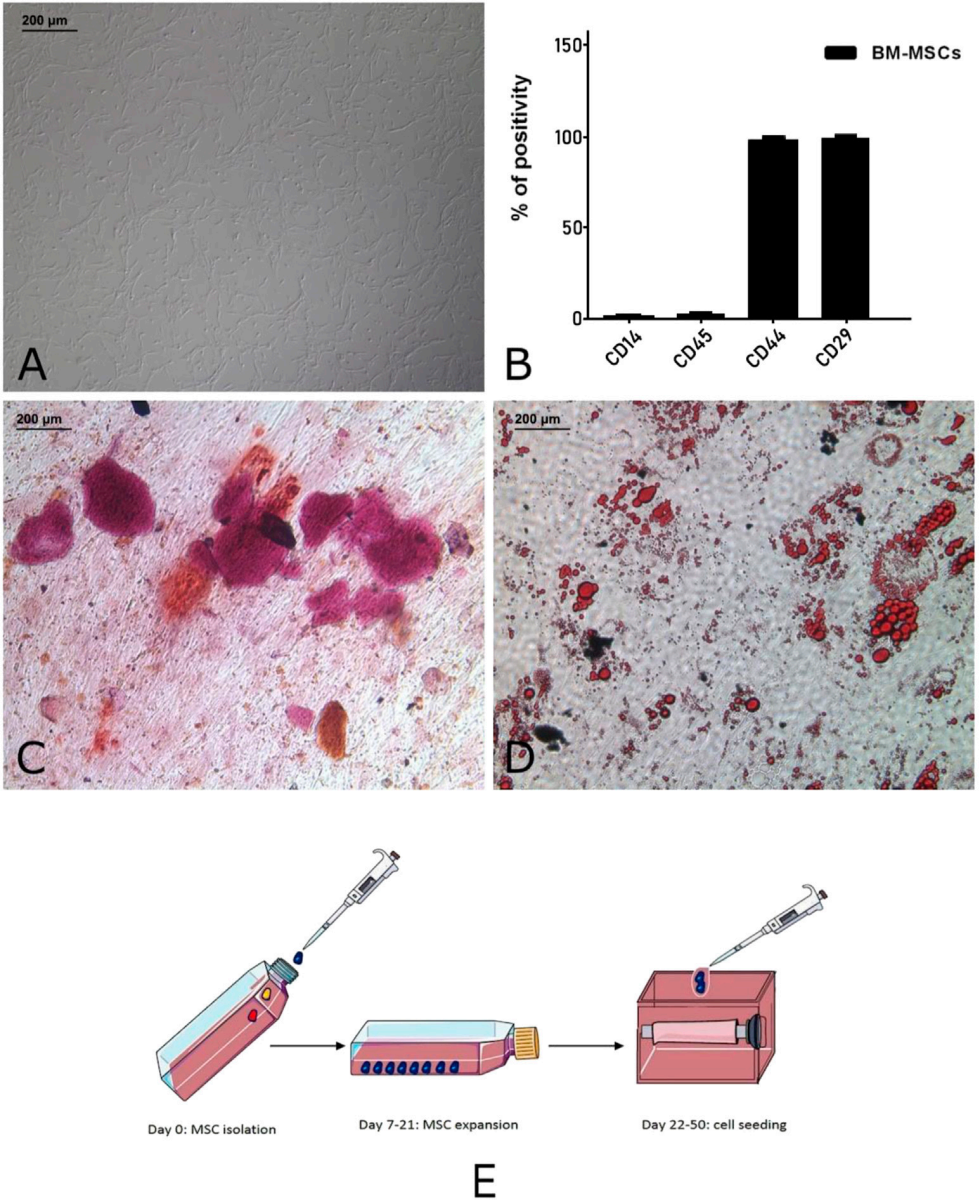
Autologous pig BM-MSC cultures (replicates: 10, corresponding to the receiving animals of the experiment). These cells displayed the characteristic spindle shaped morphology **(A)** and marker expression (CD29 and CD44) and lack of hematopoietic markers (CD14 and CD45) **(B)**. To confirm their multipotency, the osteogenic **(C)** and adipogenic differentiation **(D)** was assessed. Graphical representation **(E)** of MSC isolation, expansion and seeding on tubular scaffold. This panel was drawn using pictures from Servier Medical Art (https://smart.servier.com).

As described in our previous work (Marzaro et al., 2020), MSCs were characterized by flow cytometry (Figure 1) and then plated in 24-well plates supplemented with the appropriate culture media to induce their osteogenic and adipogenic differentiation. Cells were maintained in these conditions for 21 days, providing two weekly medium changes. At the end of the protocol, MSCs were stained using alizarin red and oil red to highlight calcium deposition and lipid droplets, respectively.

### BM-MSCs dynamic seeding on tubular acellular scaffold

The tubular decellularized scaffold was re-equilibrated in culture medium and immobilized on cylindrical support suitable for liquid passage. The scaffold was then located in a sterile chamber, connected to a vacuum pump, and seeded with MSCs previously resuspended in culture medium at the concentration of 10 × 10^6^/ml. In order to perfuse the whole scaffold during the seeding procedure, a dedicated bioreactor was conceived to realize a privileged flow of the culture medium and cells through the microchannels from the outer surface to the luminal aspect. After 2 h, enough medium was added to cover the scaffold and the culture was extended for 30 days, performing 4 re-seeding and providing two weekly medium changes. 10 μm thick slices were obtained through the Leica 1860 cryostat. Nuclei staining with DAPI nuclear marker (conc 1: 1000, Life Technologies) were performed.

### Surgery

Animals were prepared for surgery and anesthetized as previously described for the sample procurement, and anesthesia was maintained through isoflurane 1.3%–3% administration by inhalation. Intraoperative analgesia was obtained by fentanyl 2–20 μg/kg/h (IV), antibiotic coverage was guaranteed by cefazolin 25 mg/kg (IV) and omeprazole 0.7–1 mg/kg (IV) was administered at the same time.

For the scaffold implantation 10 minipigs were used because of their slower growth curve (compared to traditional large size pigs (Kang et al., 2012; Hyunwoong, 2021)), which more closely follow the growth of newborns. They were divided into 2 groups of 5 animals each, the second with different and improved techniques in gastrostomy and endoluminal stent securing. All animals were placed in left lateral decubitus and underwent a right thoracotomy, the posterior mediastinum was reached through an extrapleural way, as used in an esophageal atresia operation on a neonate, and the thoracic esophagus was exposed. A 4 cm long tract of the esophagus was removed and the gap was substituted with the full-thickness tubular scaffold of the same length previously decellularized, microperforated by QMR treatment and seeded with autologous MSCs cultures. Two different 6.0 running sutures for the mucosal and the muscular layers were used, on both upper and lower sides. The muscular layers of the native esophagus and scaffold were approximated exactly in an end-to-end fashion to create a close contact between the bleeding section of the native esophagus and the same layer of the scaffold. The mucosal and submucosal layers were easier to anastomose because of their natural elasticity on both the native and the scaffold sides. No pedicled omental flap was used to increase the scaffold vascularization, no thoracic drainage was used and a stent was positioned to keep the esophageal lumen open. A gastrostomy was added for the alimentation during the first follow up time, while the stent was secured on the upper portion through a cervicotomy on the right lateral pharyngeal side and on the lower one at the gastrostomy site.

### Follow-Up

Housed animals underwent a 6-month follow-up based on clinical controls evaluating behavior and weight gain, food intake, dysphagia, regurgitation or vomiting, monthly upper endoscopy and contrast study of the esophagus by injecting the contrast through the operative channel of the endoscope. Both the examinations were performed under general anesthesia with the same procedure of surgery. Animals were fed through the gastrostomy during the first postoperative week, then were let gradually free on oral feeding starting with liquids. At the end of the follow-up they were sacrificed to go on autopsy by intravenous injection of Tanax (MI, Italy) 3 ml/10 kg. The esophagus was removed through the previous thoracotomy and the substituted tract identified and collected for histological examination together with the anastomotic sites.

## Results

### Macroscopic and Microscopic Aspect

**FIGURE 2:**
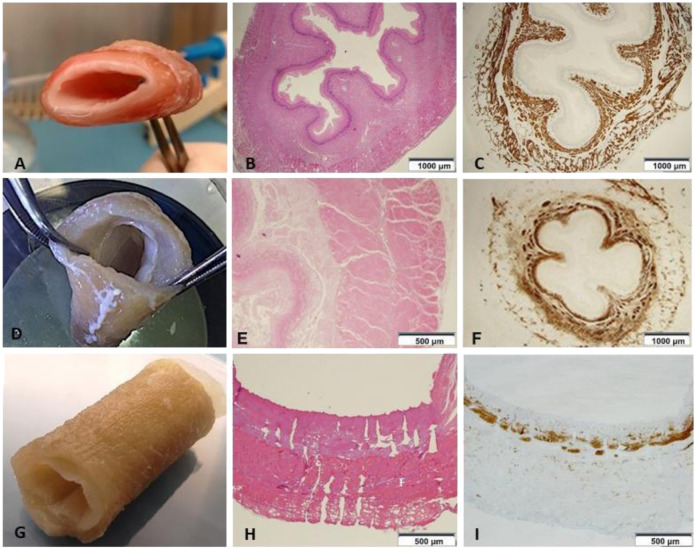
**(A)** Native esophagus. **(B)** Histology shows the preserved mucosa, submucosa and muscle layer (HE 1.25x) (*n* = 1). **(C)** Immunostaining with anti-Smooth Muscle Actin (SMA) depicting the cytoskeleton shows the normal representation of the muscle component both in the muscularis mucosae and in the muscle layer (anti-SMA 1.25x) (*n* = 1). **(D)** Esophageal scaffold macroscopic appearance after decellularization. **(E)** The wall architecture is preserved, but poor in vital cells (HE 4×) (*n* = 1). **(F)** Immunohistochemistry highlights that smooth muscle cells are significantly reduced in number and fascicles thickness (anti-SMA 1.25×) (*n* = 1). **(G)** Esophageal scaffold macroscopic appearance after decellularization and perforation with QMR technology. **(H)** On histological examination the channels from the external muscular surface to the internal mucosal layer are evident. The picture shows the channels not everywhere due to the cutting surface section passing often in the intact ECM between the channels without including all the perforations present on the same line (HE 4×) (*n* = 1). **(I)** The scaffold shows only isolated and irregularly arranged muscle bundles, which are present in the muscularis mucosae as cytoskeleton residuals, but are almost totally absent in the muscle layer (anti-SMA 4×) (*n* = 1).

Compared to the fresh material (Figure 2A), decellularized non-perforated esophagi (Figure 2D) appeared pale and translucent. This step of the procedure was characterized by a slight liquid retention with associated relative increase in thickness, general dimensions and original structure were preserved but histological examination showed the maintenance of the pink eosinophilic staining typical of collagen in the native samples (Figure 2B) also in the decellularized scaffolds (Figure 2E). Compared to the native esophagus (Figure 2C), the double-layered muscular wall was evident with the original muscle fibers disposition, as well as the mucosa and submucosa layers with strong expression of smooth muscle actin (SMA) (Figure 2F).

**FIGURE 3:**
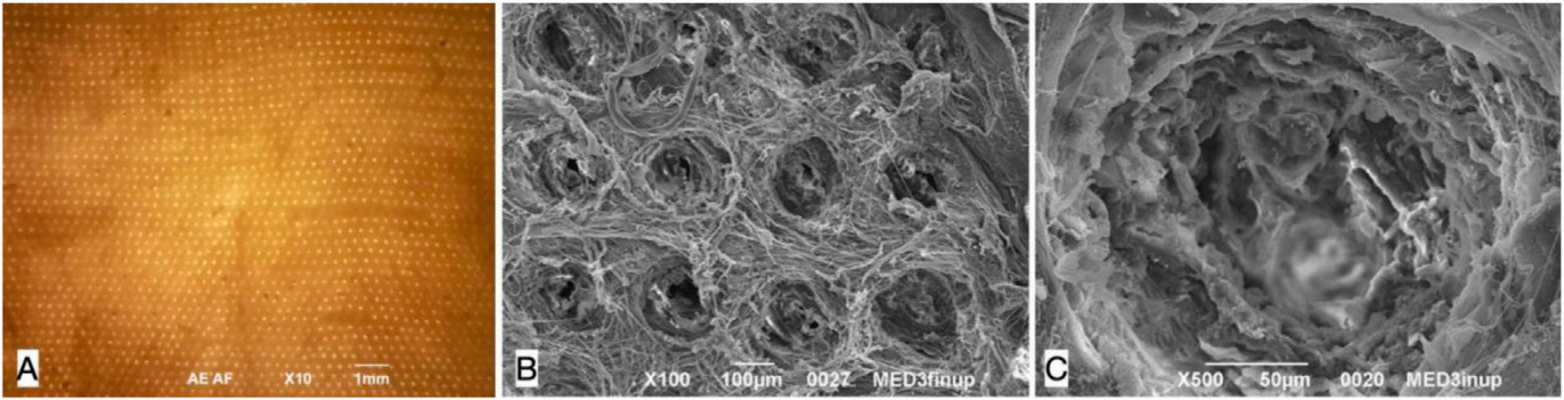
Decellularized pig esophageal scaffold after perforation: the visible surface is the esophageal muscle layer where the needle entering points for perforation are present, the mucosal layer is underlying. **(A)** Microscopic scaffold appearance after QMR: a regular microperforation pattern is appreciable (magnification: 10×, scale bar 1 mm) (*n* = 10). **(B)** SEM image of the decellularized scaffold after QMR treatment with regular microperforation pattern: interspaces between channels have the same dimension of channels diameter (magnification: 100×, scale bar 100 μm) (*n* = 10). **(C)** SEM image of a single microchannel: the ECM inside the channel is intact since no burning phenomena take palace to cause coagulation or vitrification (magnification: 500x, scale bar 50 μm) (*n* = 10).

After perforation, esophageal scaffolds appeared less pale and their thickness slightly decreased because of the compression caused by the needle action on the connective tissue: their macroscopic aspect still remained quite similar to the native esophagus (Figure 2G) since the original layers of the esophageal wall were clearly distinguishable, from the outer muscular layer to the inner mucosal one **(** Figures 2H,I **)**. The perforation procedure produced about 1,000 micro channels/cm^2^ in a regular distribution throughout the scaffold (Figure 3A). The diameter of the channels ranged from 50 to 100 μm with regular interspatial distances of the same magnitude (Figures 3B,C).

At the end of the whole procedure, we obtained a 4 cm long engineered esophagus ready for homologous MSCs seeding followed by implantation in the mini-pig model.

### Decellularization

**FIGURE 4:**
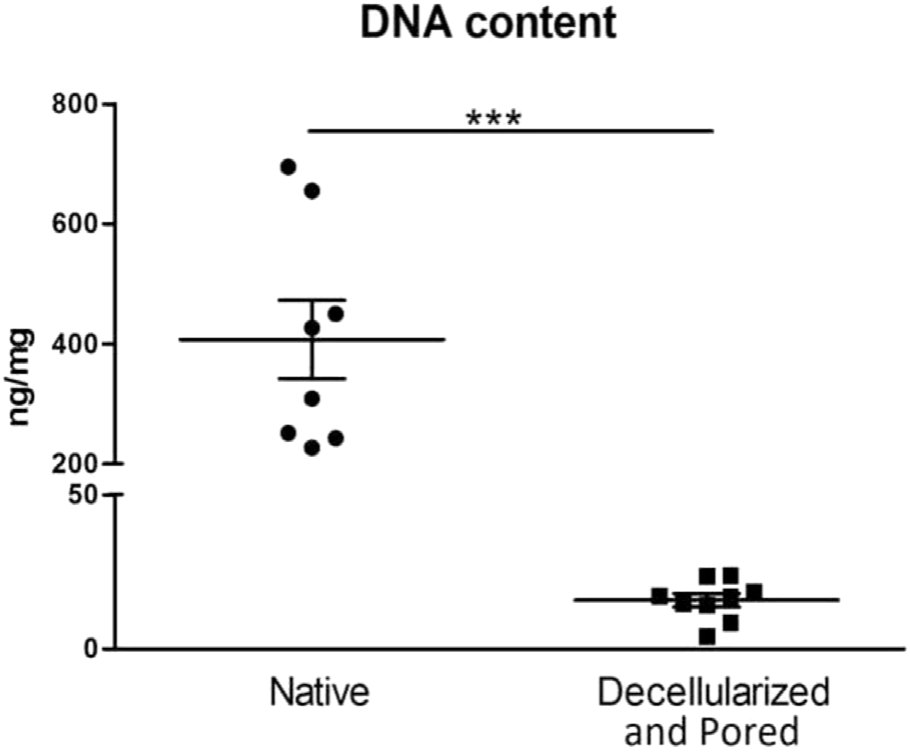
Comparison of DNA content between native samples (left column, *n* = 8) and scaffolds that underwent the decellularization and perforation procedures (right column, *n* = 9). ***p ≤ 0.001.

We compared the residual DNA amount difference between native, decellularized and perforated tissues. Scaffolds were considered efficiently decellularized when the DNA content was <50 ng/mg dry ECM, as suggested in literature (Crapo et al., 2011). In all decellularized and perforated scaffolds the DNA content was well below the threshold and significantly lower with respect to fresh esophagi, demonstrating an efficient decellularization in all samples. In particular, fresh esophagi had a median DNA content of 407.23 ng/mg dry ECM, while decellularized samples showed a median value of 15.83 ng/mg dry ECM (3.89% with respect to the native esophagus) (Figure 4).

### Comparison of extracellular matrix of native, decellularized and QMR-TREATED esophageal scaffolds by immunofluorescence analyses

**FIGURE 5:**
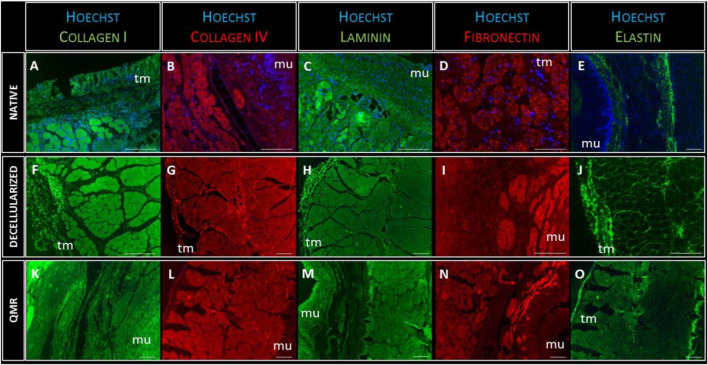
Representative images of Collagen-I (green) expression in native **(A)**, decellularized **(F)** and QMR-treated esophagi **(K)**; collagen-IV (red) expression in native **(B)**, decellularized **(G)** and QMR-treated esophagi **(L)**; laminin (green) expression in native **(C)**, decellularized **(H)** and QMR-treated esophagi **(M)**; fibronectin (red) expression in native **(D)**, decellularized **(I)** and QMR-treated esophagi **(N)**; elastin (green) expression in native **(E)**, decellularized **(J)** and QMR-treated esophagi **(O)**. Blue: nuclei staining with Hoechst. Mu: mucosa; tm: tunica muscularis. Native esophagi *n* = 1, decellularized esophagi *n* = 1, QMR-treated decellularized esophagi *n* = 2. Scale bar: 200 µm.

The presence of nuclei was investigated by Hoechst staining, and the presence of five essential ECM proteins was detected: Laminin, Fibronectin, Elastin, Collagen I and IV. These proteins are involved in preserving the structural characteristics of the esophageal scaffold and its capacity to properly interact with the cells (Figure 5).

The immunofluorescence staining confirmed the complete removal of nuclear remnants from both decellularized and QMR treated scaffolds. Cells nuclei (blue staining) were only visible in native samples, to confirm the results from the DNA analysis. The expression of each of the five investigated proteins looked steady in native, decellularized and perforated samples. Immunofluorescence analysis gave evidence that the ECM composition was not altered due to the different steps of scaffold preparation since the fundamental proteins are still properly present.

### Biomechanical Results

**FIGURE 6:**
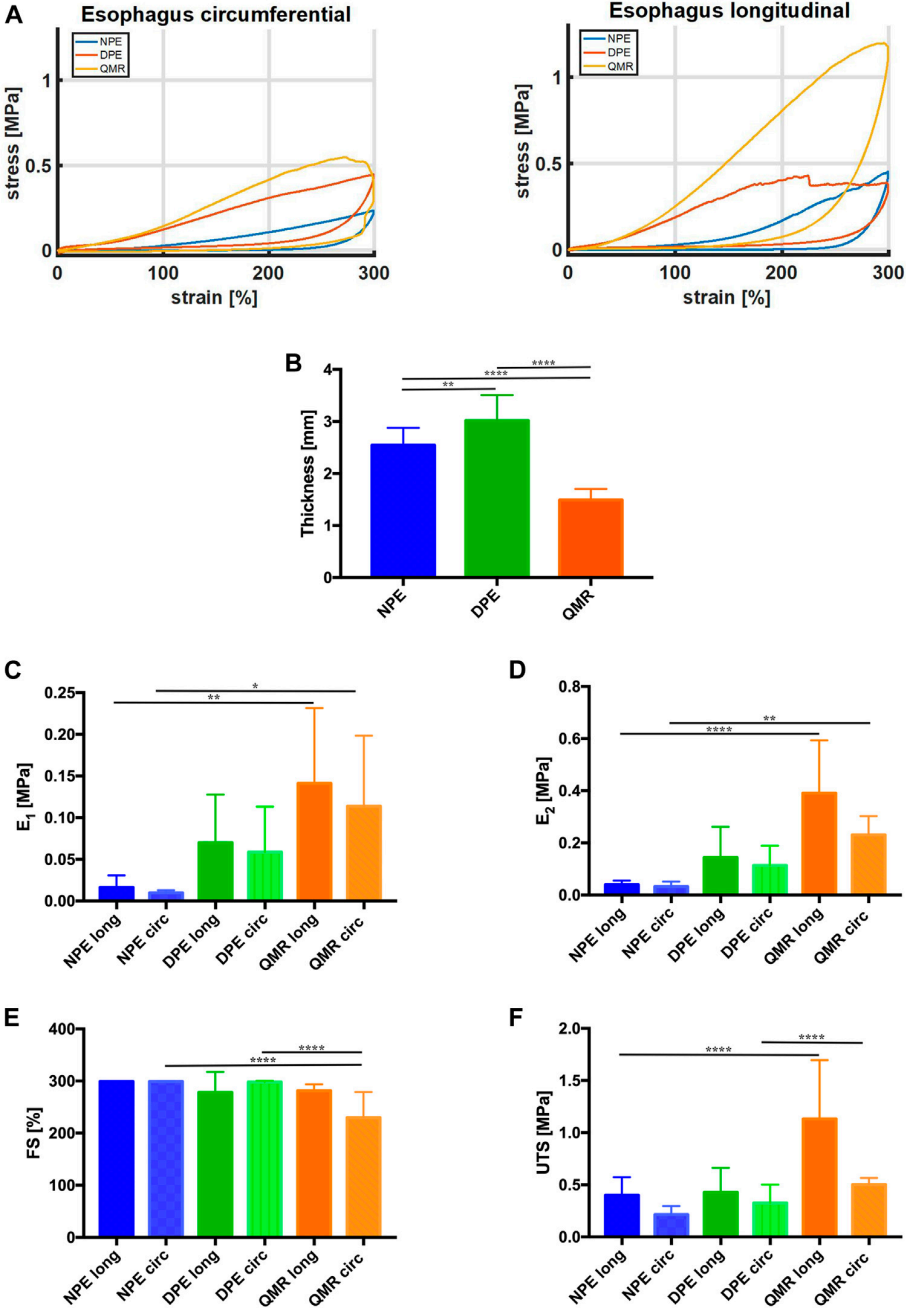
**(A)** Representative stress-strain curves measured from the investigated tissues along both longitudinal and circumferential directions. **(B)** Thickness values (mm) of longitudinal and circumferential NPE, DPE, and QMR samples. **(C)** E_1_ values (MPa) and **(D)** E_2_ values (MPa). **(E)** FS values (%) and **(F)** UTS values (MPa). For each kind of tissue, *n* = 8 samples were considered along longitudinal direction, *n* = 8 samples along circumferential direction. Significance difference was set as it follows: *p < 0.05; **p < 0.01; ****p < 0.0001.

Representative stress-strain curves measured during tensile tests from the investigated tissues along both longitudinal and circumferential directions are depicted in Figure 6A. It is likely to observe that QMR samples show a distinct behavior depending on the considered direction: along the longitudinal direction, they do achieve stress values much higher than along the circumferential direction.

Figure 6B compares the thickness of native (NPE), decellularized (DPE) and QMR-treated samples (QMR): the decellularization procedure causes a significant increase in thickness (from 2.544 ± 0.33 mm to 3.018 ± 0.49 mm), which significantly decreases after perforation (1.49 ± 0.21 mm).

The Young’s modulus E1 (Figure 6C) progressively increases for both longitudinal and circumferential directions going from NPE to DPE and QMR. It is worthy to notice that circumferential samples always exhibit lower values with respect to their longitudinal counterparts. In detail, E1 for longitudinal NPE is 0.016 ± 0.014 MPa against 0.009 ± 0.003 MPa circumferential; longitudinal DPE is 0.07 ± 0.06 MPa against 0.058 ± 0.06 MPa circumferential; longitudinal QMR is 0.014 ± 0.09 MPa against 0.11 ± 0.08 MPa circumferential.

The E2 modulus (Figure 6D) shows a similar trend but with higher values than modulus E1. E2 for longitudinal NPE is 0.044 ± 0.015 MPa against 0.033 ± 0.02 MPa circumferential; longitudinal DPE is 0.144 ± 0.12 MPa against 0.114 ± 0.07 MPa circumferential; longitudinal QMR is 0.39 ± 0.21 MPa against 0.23 ± 0.07 MPa circumferential. Generally, samples in the longitudinal direction are stiffer than in the circumferential direction.

Figure 6E compares the FS values of the investigated samples. Three of them withstand the imposed 300% deformation, which is much higher than the physiological one: they are circumferential DPE, longitudinal and circumferential NPE. This evidence confirms that the lower is the stiffness of the samples, the higher is the failure strain. Perforation results in decreased FS values: 281.4 ± 12.51.

UTS values agree with the trend registered for both E1 and E2 (Figure 6F). Circumferential samples develop UTS values (NPE = 0.213 ± 0.08 MPa, DPE = 0.325 ± 0.18 MPa, QMR = 0.502 ± 0.06 MPa), which are lower than the longitudinal counterparts (NPE = 0.401 ± 0.17 MPa, DPE = 0.427 ± 0.23 MPa, QMR1.13 ± 0.56 MPa).

The biomechanical results confirmed the anisotropic behavior of the investigated tissues (Sommer et al., 2013; Mir et al., 2016), which exhibit distinct mechanical features along the longitudinal and the circumferential directions. Anisotropy is maintained for both small and large deformations: this is confirmed by the trend of E1 and E2 moduli.

### Estimation of the increase in cell seeding available area

QMR microperforation allowed creating microchannels within the esophageal scaffolds. As a consequence, if compared to a non-perforated scaffold, the available surface for MSCs seeding was increased due to the new spaces created inside the connective tissue. This is directly related to the number, diameter and depth of the channels. Actually, the needles we used are cone-shaped only on the tip and cylindrical on the rest. Then a possible estimation of the perforation results was assumed to be mainly cylindrical and cone-shaped on the tip.

Hypothesis: 1000 micro channels/cm^2^ → Density = 10 micro channels/mm^2^ Analyzed 2D surface (S_2D_): 1 mm^2^ Micro channel diameter (D_CHANNEL_): 0.1 mm Microchannel deepness (L_CHANNEL_): 1.5 mm (same as micro channeled scaffold thickness)

Assessment:

1 mm cylindrical channels with 0.5 mm cone-shaped tip
Cylindrical body (LBODY = 1 mm)
Cone tip (LTIP = 0.5 mm)

Surface computation:

Microchannel cylindrical lateral surface = SLBODY = LBODY x π x D_CHANNEL_ = 0.314 mm^2^
Microchannel cone tip lateral surface = S_LTIP_ = L_TIP_ x π x D_CHANNEL_ = 0.0785 mm^2^

Total microchannel lateral surface

S_CHANNEL_ = S_LBODY_ + S_LTIP_ = 0,314 mm^2^ + 0,0785 mm^2^ = 0,3925 mm^2^. In 1 mm^2^ there are 10 microchannels → S_INCREASE_ = Density x S_CHANNEL_ = 3,925 mm^2^

Total residual upper planar surface:

S_RES_ = S_2D_ - Density x microchannel base area = 1 – Density x π x (D_CHANNEL_/2)^2^ = 0,9215 mm^2^

Total 3D surface for seeding

S_3D_ = S_INCREASE_ + S_RES_ = 3,925 mm^2^ + 0,9215 mm^2^ = 4,8465 mm^2^

Estimated proportional surface increase for cell seeding:

S_3D_ / S_2D_ x 100 = **484%**

### BM-MSCS seeding in dynamic condition

**FIGURE 7:**
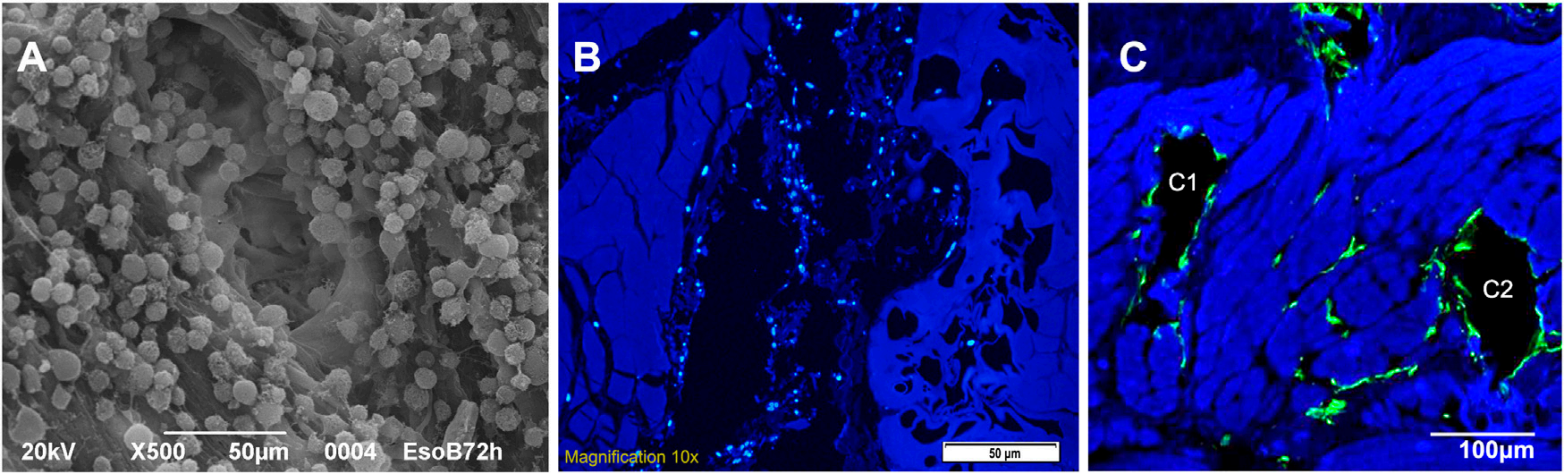
**(A)** SEM image of a single microchannel from the needle entering point after seeding (A1): it shows the regular adhesion of cells on the microchannel walls (magnification: 500x, scale bar 50 μm). **(B)** Autologous pig MSCs culture (DAPI nuclei coloration): cells are seeded inside the channel (B1, central black cavity on longitudinal section) but nuclei are also visible out of the major cavity through the ECM leakages close to the channel (scale bar 50 μm). **(C)** Autologous pig MSCs culture (F actin, green coloration): cells appear adhered to the walls of the channels (C1 - C2, two black cavities on transversal section) but they are also spreading out of them inside the ECM leakages between and close to the channels (scale bar 100 μm).

Tubular scaffolds were cultured for 30 days in dynamic conditions because of the high 3D complexity (as graphically resumed in Figure 1E) given by their architecture and the presence of all the esophageal wall layers. They underwent 3 re-seeding procedures in order to maximize the contact between the cells and the scaffolds, and were microscopically analyzed at the end of the incubation period to evaluate their vitality. We had a demonstration of the cell’s good behavior since they successfully adhered to the surface of tubular scaffolds and easily penetrated the microchannels, as shown by SEM images where adherent cells are visible in their inner parts (Figure 7A). Moreover, at the end of seeding, the migration of MSCs outside the channels and between the ECM fibers was observed (Figures 7B,C). We guess that this is due to the prolonged culturing time and re-seedings which maximized the cells potential to spread throughout the connective tissue exploiting the open leakages on the microchannel’s walls.

### Surgery

At surgery, the scaffold was pliable but resistant, and two different termino-terminal (TT) anastomosis were performed on the mucosal and muscular layers, on the upper and on the lower pole, juxtaposing the bleeding esophageal edges of the native esophagus to the ones of the seeded scaffold with running absorbable 5.0 stitches. It was possible to take advantage of the natural elasticity of mucosa and submucosa on both the native esophagus and the scaffold, while the muscular layers were thicker and stiffer. The bleeding support of the native esophagus was clear because at the end of the procedure the scaffold acquired a reddish appearance (Supplementary Figure S1).

### Follow-Up

Postoperative follow-up was particularly hard-working because of the animals’ repetitive attempts to remove the esophageal stent and the gastrostomy device. In the first group of 5 minipigs, many issues related to gastrostomy and stent securing were encountered and unfortunately resulted in 4 animals suffering from major abdominal and neck complications that led them to death. In these cases it was not possible to do the autopsies. In the second 5 animals’ group we changed the gastrostomy and esophageal stent technique and only one of the animals did not survive because of the same problems. In this case the autopsy confirmed that the scaffold was still anastomosed. A total of 5 animals (1 in the first group, 4 in the second group) successfully completed the full 6-months follow-up period. As evidenced by radiologic and endoscopic controls (Supplementary Figure S2) the anastomosis did not show leakages and the mucosa did not show interruption corresponding to the substituted tract, the animals received liquid food in the first postoperative week while nutritional support was assured by gastrostomy, then were let free on oral feeding. Because of the frequent removals, an average of 10 endoscopic esophageal dilatations with stent repositioning (range 6–19) were done to preserve the patency of the esophageal lumen. The longer the period with stable esophageal stent, the lesser was the need for esophageal dilatation. In two cases a re-operation has been necessary because of the removal of the gastrostomy tube, in one of them also caused by a subcutaneous abscess close to the gastrostomy site. The median period between two dilatations was 69.5 days (range 28–105).

### Histological results after implantation

**FIGURE 8:**
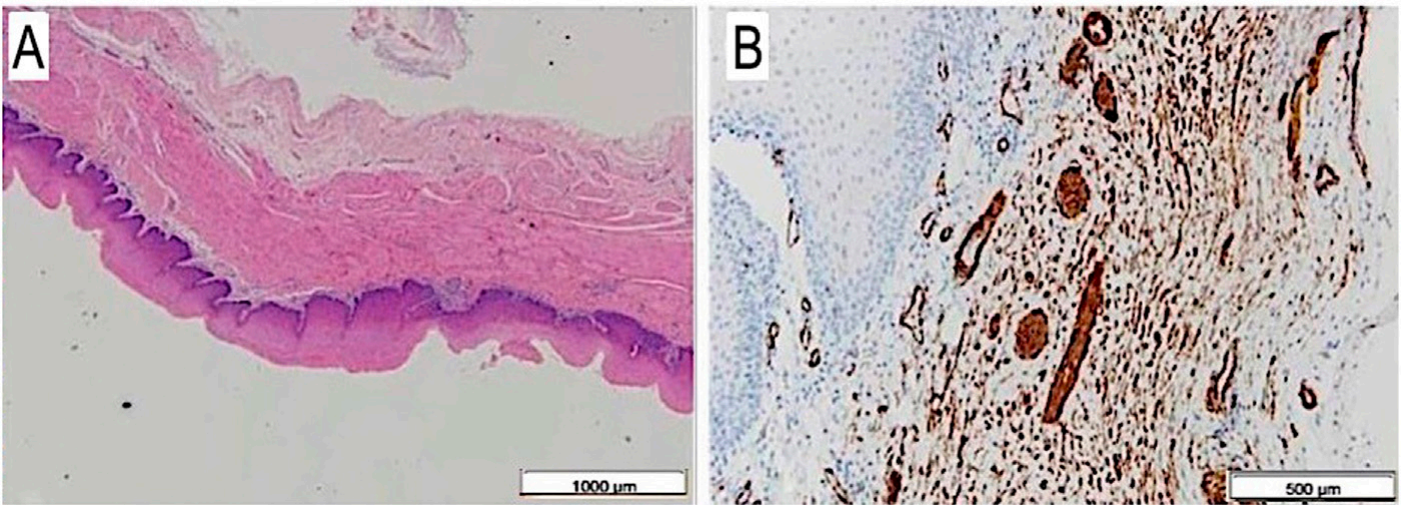
**(A)** Full thickness esophageal wall scaffold on histology after 6 months from surgery: mucosa, submucosa and muscular layer are well visible, H&E, 1.25×. **(B)** The same on anti-SMA: single and thin muscular fibers, also with vascular images, at 6 months autopsy, Anti-SMA, 4×.

The autoptic examination demonstrated intact scaffold anastomoses with their full-thickness regeneration reproducing all the corresponding layers of the native esophagus it was anastomosed to. Actin-and desmin positive cells were observed throughout the entire muscular layer resembling a neo-muscle wall. Moreover, a complete mucosal regeneration was evidenced and a new submucosal layer with vascular infiltration was present, as in the original organ (Figures 8A,B).

## Discussion

Tissue engineered tubular organ replacement can be obtained from artificial or natural scaffolds. Artificial biomaterials are tunable towards the desired shape and dimension, according to the organ to be realized; they are the result of complex industrial synthetic processes, and their adjustable stiffness make them an optimal choice for organ integration or regeneration. On the other hand, they lack the biochemical signals that naturally guide cells’ functions: thus, they do not have the potential for natural cellular repopulation and show a host response different from natural biomaterials (Mir et al., 2016).

Natural biomaterials can be obtained from biological organs after the removal of nuclear and cellular components by a sequence of relatively cheap treatments. Notably, the procedure we used assured optimal results in terms of both residual DNA content and preservation of the original tissue structure (Sommer et al., 2013). The residual ECM is biodegradable and disappears in a few weeks (Sadtler et al., 2019), is biologically compatible and does not produce toxic, injurious, carcinogenic, or immunological response in living tissue even if xenogeneic (BaguneidSeifalian et al., 2006; MaeleneWong et al., 2016). Moreover, it stimulates vascular ingrowth (Fallas et al., 2018), cell adhesion (one of the major issues with artificial scaffolds), growth, proliferation and differentiation, diffusion and survival (Voigt et al., 2001; GeorgeHussey et al., 2018; García-Gareta et al., 2020), resembling the native organ so closely that decellularized scaffolds have been considered the best memory of the original organ.

For all these reasons, natural acellular scaffolds based on native ECM have been proposed as the ideal materials for esophageal repair (Hussey et al., 2017), but it is assumed that they cannot allow long full-thickness circumferential reconstructions unless modified to be added with cells and liquids (Totonelli, 2012).

Decellularized tissues/organs do not release toxic byproducts due to their natural degradation, and do not stimulate foreign body reaction unless they are ineffectively decellularized or are chemically cross-linked (Anderson et al., 2008; Totonelli, 2012). Above all, they resemble the native organ macro- and microscopically, preserving also the biochemical signals essential for cell adhesion/proliferation. They have been also investigated, and sometimes clinically used, for their potential effects in downregulating the expression of neoplastic cell populations (Badylak et al., 2011; Saldin et al., 2019; Naranjo et al., 2020). ECM complex composition is strictly specific for each organ, which hinders artificially creating a scaffold able to fully mimic both structure and functions of native ECM (Chan and Leong, 2008). Finally, the composition of mammalian ECM is similar among different species. Therefore, the host reaction to implanted natural acellular matrices is similar to both xenogeneic and allogeneic scaffolds (Keane and Badilak, 2015).

In conclusion, all the above-mentioned issues allow assuming that a scaffold with the preferred ECM components would be considered the preferred TE matrix (Keane et al., 2015). On the other hand, natural biomaterials are not tunable with regard to shape and dimensions unlike artificial ones.

Many efforts have been made towards establishing standardized criteria for a successful decellularization (Crapo et al., 2011), but little has been considered in terms of function of a decellularized scaffold after implantation.

Porosity is probably the most important issue for *in vivo* vascular and cellular integration. It is essential for cell nutrition, proliferation and migration during tissue regeneration. It has been noticed for mechanical interlocking with the surrounding tissue, guiding the hosts vascular, neurogenic and stem cell infiltration and releasing biochemical factors like proteins or genes providing good substrates for nutrient exchange (Hollister, 2005; Loh and Choong, 2013; Zhu et al., 2019). Moreover, the scaffold mean pore size influences cells’ proliferation and differentiation in some specific organogenesis situations (Matsiko et al., 2015). Finally, porosity is essential for a 3D cell seeding inside the scaffold, definitely the best environment for an optimal immunomodulatory and regenerative cell-cell-scaffold-host interactions (Jeong et al., 2020).

Unfortunately, porosity is rather different between natural and artificial biomaterials. Artificial biomaterials are composed - almost invariably - of synthetic fibers that privilege the spaces between one and another, or solid materials with regular cavities. On the contrary, natural biomaterials based on muscular tissue privilege the purity of the connective tissue, which is completely respectful of the original anatomy but do not allow a deep cell penetration since residual ECM lacks adequate porosity.

Tubular-shaped constructs are difficult to be realized (H.Nam et al., 2020), and many biomaterials have been proposed as scaffolds to promote esophageal tissue engineering reconstruction. Among them, gastric acellular matrices (Urita et al., 2007), AlloDerm^®^ (Isch et al., 2001), xenogeneic small intestinal submucosa (SIS) and urinary porcine bladder matrix (UBM) (Badylak et al., 2000; Badylak et al., 2005; Poghosyan et al., 2015; Catry et al., 2017), porcine aorta (Kajitani et al., 2001), esophageal acellular matrices (Marzaro et al., 2006), retrievable synthetic scaffold carrying autologous cells (La Francesca et al., 2018; Nam et al., 2020), mixed materials with bioinks (Kajitani et al., 2001), PLC/PLGA (Polycaprolactone/poly(lactic-co-glycolic) acid) tubular scaffolds (Jensen et al., 2015), non-absorbable materials such as polyethylene terephthalate, polyurethane electro-spun scaffold (Miki et al., 1999), and silicone or expanded polytetrafluoroethylene (Gonzalez Saez et al., 2003). Above all, the use of pre-seeded scaffolds for full-circumferential esophageal reconstruction resulted in higher degree of regeneration and lower inflammation rates with respect to scaffolds implanted alone (Tan et al., 2013). It is worth mentioning that artificial biomaterials stimulate non-regenerative immune responses, opposite to the natural ones that are characterized by M2/Th2 reconstructive reaction (Mariani et al., 2019).

Finally, only episodic successful cases of commercial biomaterials used in human patients to repair esophageal iatrogenic lesions have been reported (Reames and Lin, 2013; Nieponice et al., 2014; Dua et al., 2016).

Our group previously tested a homologous acellular esophageal muscular scaffold as a partial substitute of the esophageal muscle layer (Marzaro et al., 2006). A patch of homologous non-perforated smooth muscle scaffold, decellularized with the Meezan protocol (Meezan et al., 1975; Badylak and Gilbert, 2008), was used to cover a defect in the muscular layer alone of the thoracic esophagus in 3 months old pigs. The mucosal layer was left intact to allow autonomous oral feeding. Non-seeded patches showed a more severe inflammatory response and were negative for α-smooth muscle actin immunostaining 3 weeks after surgery, while the patches seeded with autologous satellite cells previously isolated from the cervical esophagus had a consensual growth with the host without dysphagia or stenosis, with vascular and small fascicules muscular ingrowth. Unfortunately, the 2D limited number of living cells seeded on the scaffold upper surface compromised the reconstructive reaction in some cases.

For this reason, we moved toward the actual microperforated donor derived decellularized homologous esophageal scaffold. The perforation of the decellularized esophageal scaffold opened new channels by selectively breaking part of the ECM protein bindings. As already shown in our previous study (Marzaro et al., 2020), and already mentioned above, we realized about 1,000 micro channels/cm^2^ in a regular distribution throughout the scaffold, pore diameters ranged from 50 to 100 μm and interspaces had the same dimensions (Figure 3).

In the present application, the tubular shape of the esophagus was preserved as well as all the original esophageal wall layers. Moreover, the decellularization method was performed through a new custom-made perfusion bioreactor.

The scaffold macroscopic appearance was preserved while thickness was slightly reduced if compared to the decellularized scaffold due to the partial compression on the ECM structure by the needle action; no burns or tissue damages were documented, both macroscopically and microscopically (analysis performed with a digital microscope and a Scanning Electron Microscopy). The resulting scaffold looks like an evolved natural one with a controlled porosity. Since the microchannels penetrate from the outer muscular surface to the submucosa and mucosa layers, the most important effect is due to the substantial increase of the available surface for cell seeding (e.g., + 400%). This is of the utmost importance because, owing to this artificial porosity, we can hypothetically avoid cell dispersion during seeding and increase their number and interaction inside the connective tissue.

At the same time, we can claim that the cells seeded inside the channels are close and communicate to each other, but are also able to spread outside and migrate into the surrounding tissue through the leakages of the ECM fibers that are still present inside the microchannels since, as evidenced before, the perforation does not provoke burning or coagulation on the channel’s wall (Figure 7). Vascular structures of the scaffold, located between tunica mucosa and muscularis, are maintained. The closure at the very beginning of the channels, at the surface proximity, is due to the sample crushing during sectioning.

Histology confirmed an improved MSC 3D dispersion, if compared to the 2D seeding documented in our previous experimental trial, since we found nuclei and cytoplasm inside all the scaffold layers, from the outer surface to the mucosa. Cells also spread out the microchannels through the leakage of the ECM fibers that still communicate with the pores after perforation.

Due to the perforation treatment, another important side effect is the improved stiffness of the perforated esophagus if compared to native and decellularized ones. The needle action during the perforations induces partial ECM compression with thickness decrease and, consequently, higher values of both elastic modules E1 and E2 and lower values of failure strain. These characteristics were appreciated at surgery: the scaffold pliability and resistance made it easy to realize the anastomosis between the scaffold and the native esophagus, even using two different sutures on the mucosal and the muscular layers, naturally restoring the esophageal anatomy.

Anyhow, since the perforation is respectful and does not alter the anatomical disposition of the ECM connective fibers of every single layer, the macroscopic appearance and the dimensions of the scaffold remain the same of the original esophagus, as does the microscopic structure that still reproduces the native one.

In our model, we have shown that the seeding of autologous MSCs is able to promote the regeneration of all the scaffold layers, reproducing normal tissue histology. It is unlikely that tissue regeneration can completely derive from differentiation of seeded MSCs since it is well known that they have short *in vivo* living capability, at least when infused intravenously (Eggenhofer et al., 2012; Eggenhofer et al., 2014). The effect could be largely derived from the ability of MSCs to promote tissue regeneration from adjacent portions of healthy esophagus, although we were not able to clarify the origin of cells that repopulated the scaffold *in vivo*. Our findings are also in contrast to other attempts based on different cell populations seeded on the muscular part and the inner epithelial layer of a similar scaffold (Urbani et al., 2018). Based on this experience, further experimental trials in a mini pig animal model are currently ongoing to improve the surgical model of the esophageal substitution.

## Conclusion

Starting from an allogeneic donor-derived decellularized esophagus and through the treatment with the QMR microperforation, we obtained a microperforated decellularized scaffold whose stiffness and pliability are ideal for the surgical application. This device possesses an extremely dense full-thickness web of microchannels, which greatly increase the surface area available for cells seeding: cells can easily penetrate inside and result in a homogenous spatial distribution. Moreover, the proposed device combines the advantageous characteristics of artificial and natural scaffolds: an improved stiffness with a high porosity to facilitate cells’ distribution; an intrinsic biodegradability; the maintenance of ECM biochemical signals that guide cells’ adhesion and proliferation.

The QMR microperforation treatment produces a regular pattern of microchannels that represent a beneficial microenvironment to improve cell-cell-scaffold interactions, thus promoting the regenerative process. From the surgical point of view, the proposed device allows the easy anastomosis to the native esophagus followed by the regeneration of all esophageal wall layers. Therefore, it represents a promising tool for esophageal tissue engineering.

Further *in vivo* trials on large animal-models are in progress to assess all the properties of the microperforated tubular scaffold in esophageal reconstruction without strictures or leakage.

## Data Availability

The raw data supporting the conclusion of this article will be made available by the authors, without undue reservation.
