# Supplementary figures and images for: Decellularized esophageal tubular scaffold microperforated by quantum molecular resonance technology and seeded with mesenchymal stromal cells for tissue engineering esophageal regeneration
Source: Front Bioeng Biotechnol. 2022 Oct 4;10:912617. doi: 10.3389/fbioe.2022.912617 (PMC9576845; doi:10.3389/fbioe.2022.912617)

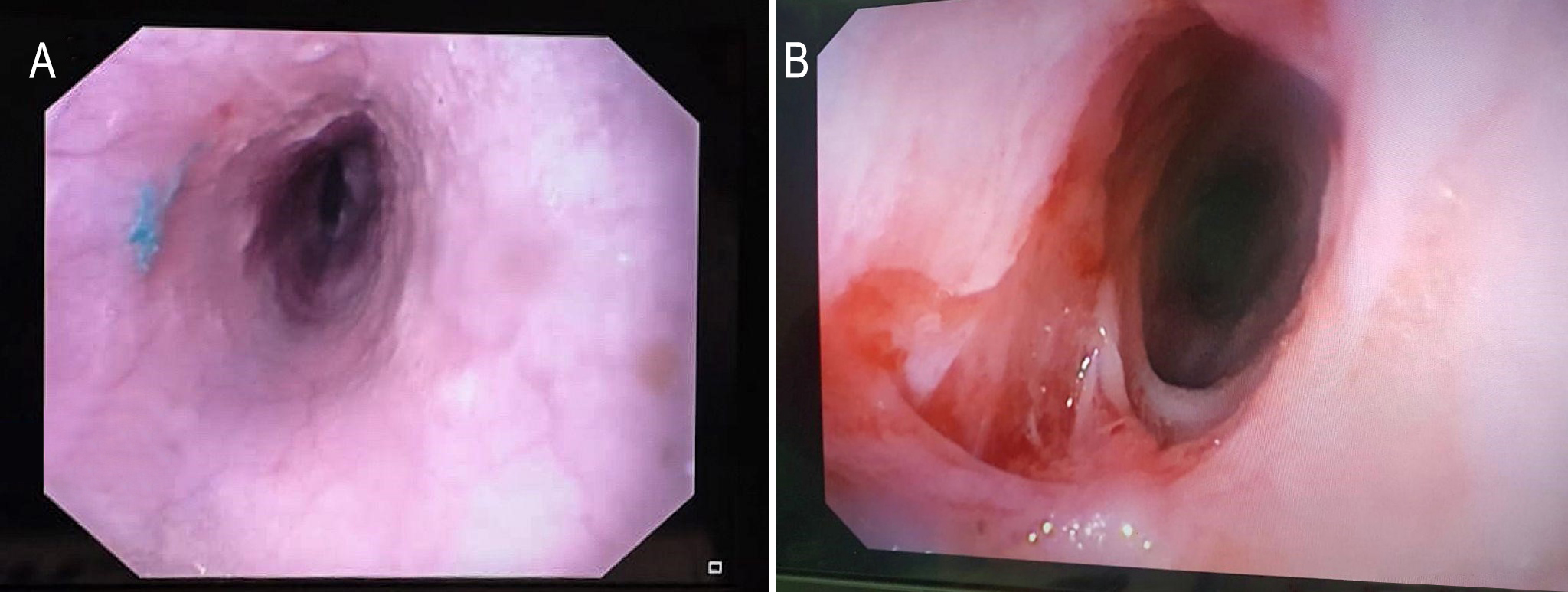

Supplement: Supplementary file 1 [file Image2.jpg]

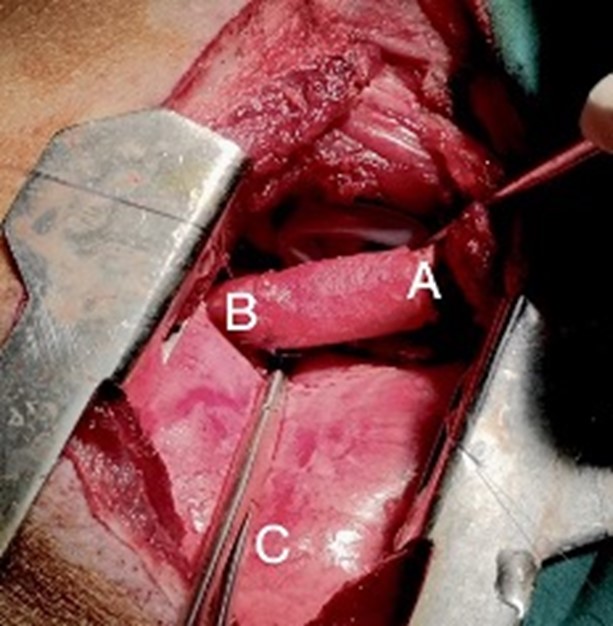

Supplement: Supplementary file 3 [file Image1.jpg]
