# Supplementary material for: Decellularized esophageal tubular scaffold microperforated by quantum molecular resonance technology and seeded with mesenchymal stromal cells for tissue engineering esophageal regeneration
Source: Front Bioeng Biotechnol. 2022 Oct 4;10:912617. doi: 10.3389/fbioe.2022.912617 (PMC9576845; doi:10.3389/fbioe.2022.912617)
Supplement: Supplementary file 2 [file DataSheet1.pdf]

Supplementary figure 2. Endoscopic appearance of a full regenerated esophageal scaffold with normal mucosa and visible residual stitch material (A), endoscopic appearance after esophageal dilatation (B).

Supplementary figure 1. Surgical implantation of the tubular microperforated esophageal scaffold. (A) Upper anastomosis. (B) Lower anastomosis. (C) Pleura.
